# Supplementary material for: Extraplexus versus intraplexus ultrasound-guided interscalene brachial plexus block for ambulatory arthroscopic shoulder surgery: A randomized controlled trial
Source: PLoS One. 2021 Feb 18;16(2):e0246792. doi: 10.1371/journal.pone.0246792 (PMC7891753; doi:10.1371/journal.pone.0246792)
Supplement: S1 File — (DOCX) [file pone.0246792.s001.docx]

**Supplemental File 1. Ultrasound images of extraplexus versus intraplexus injection.** Extraplexus injection (A) anterior and posterior to brachial plexus in the plane between the perineural sheath and scalene muscles. Intraplexus injection (B) in between C5 and C6 nerve roots.

1. Extraplexus injection


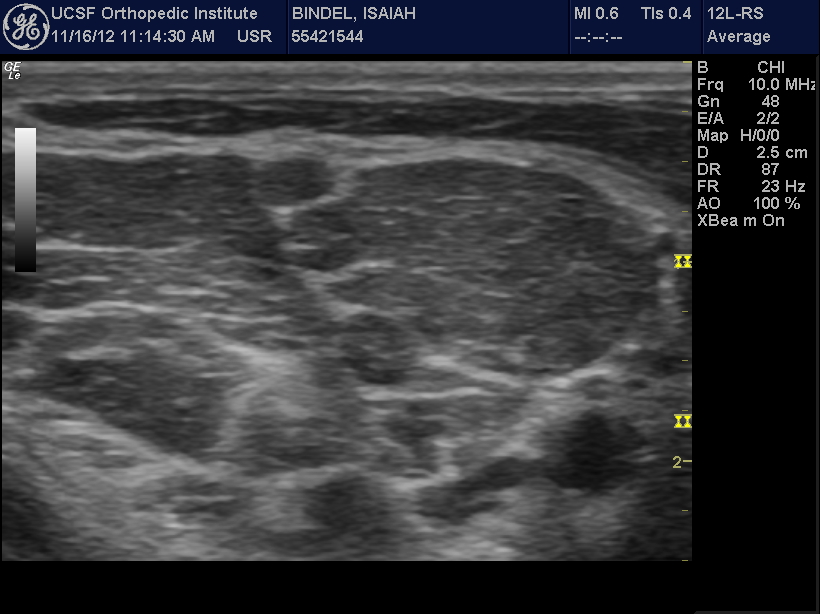


AS

MS

Anterior

Posterior

BP

1. Intraplexus injection


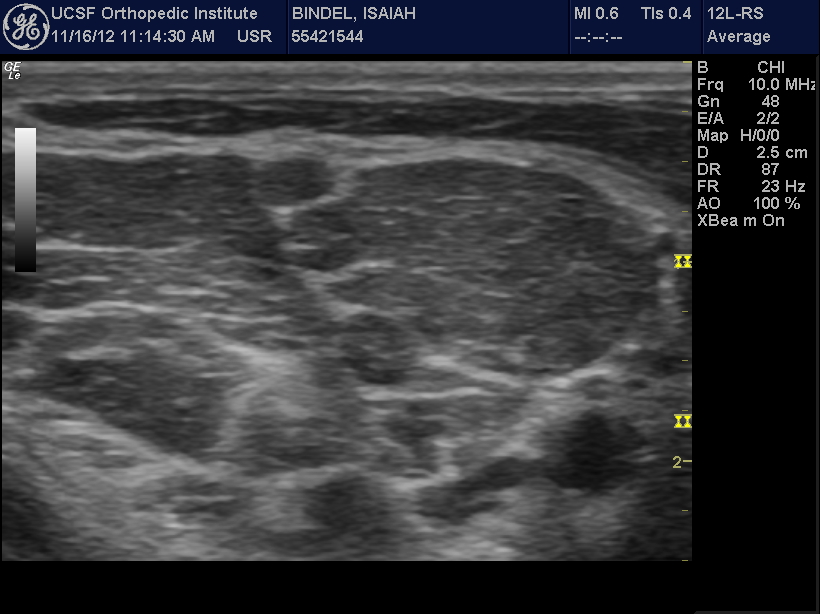


AS

MS

Anterior

Posterior

BP

AS: anterior scalene muscle, BP: brachial plexus, MS: middle scalene muscle; Arrow represents needle trajectory
