# Supplementary material for: Extraplexus versus intraplexus ultrasound-guided interscalene brachial plexus block for ambulatory arthroscopic shoulder surgery: A randomized controlled trial
Source: PLoS One. 2021 Feb 18;16(2):e0246792. doi: 10.1371/journal.pone.0246792 (PMC7891753; doi:10.1371/journal.pone.0246792)
Supplement: S2 File — (DOCX) [file pone.0246792.s002.docx]

Supporting File 2. **Oral Morphine Equivalence Conversion Equation**

Total oral morphine equivalents = [preoperative fentanyl IV (mcg) * 0.3] + [intraoperative fentanyl IV (mcg) * 0.3] + [recovery room fentanyl IV (mcg) * 0.3] + [recovery room hydromorphone IV (mg) * 20] + [recovery room hydrocodone (mg)] + [recovery room oxycodone (mg) * 1.5] + [recovery room meperidine IV (mg) * 0.3] + [Postoperative day 1 hydrocodone (mg)} + [Postoperative day 1 oxycodone (mg) * 1.5] + [Postoperative day 1 hydromorphone PO (mg) * 4] + [Postoperative day 1 morphine PO (mg)] + [Postoperative day 1 tramadol PO (mg) * 0.25] + [Postoperative day 1 codeine PO (mg) * 0.15].
