# Supplementary material for: Extraplexus versus intraplexus ultrasound-guided interscalene brachial plexus block for ambulatory arthroscopic shoulder surgery: A randomized controlled trial
Source: PLoS One. 2021 Feb 18;16(2):e0246792. doi: 10.1371/journal.pone.0246792 (PMC7891753; doi:10.1371/journal.pone.0246792)
Supplement: S5 File — (DOC) [file pone.0246792.s005.doc]

**Optimal Location of Local Anesthetic Injection for Ultrasound-guided Interscalene Nerve Block**

**Clinical Research Protocol**

**Principal Investigator/Sponsor: Pedram Aleshi, MD**

**Co-Principal Investigator: Monica Harbell, MD**

**Co-investigators: Kerstin Kolodzie, MD, Matthias Behrends, MD; Matthias Braehler, MD, PhD, Sakura Kinjo, MD**

**Protocol number:** 12-10146

[1 BACKGROUND 4](#__RefHeading___Toc262733374)

[1.1 Overview of Clinical Studies 9](#__RefHeading___Toc262733376)

[2 STUDY RATIONALE 9](#__RefHeading___Toc262733377)

[2.1 Risk / Benefit Assessment 9](#__RefHeading___Toc262733378)

[3 STUDY OBJECTIVES 9](#__RefHeading___Toc262733379)

[3.1 Primary Objective 9](#__RefHeading___Toc262733380)

[3.2 Secondary Objectives 9](#__RefHeading___Toc262733381)

[4 STUDY DESIGN 9](#__RefHeading___Toc262733382)

[4.1 Study Overview 9](#__RefHeading___Toc262733383)

[5 Criteria for evaluation 10](#__RefHeading___Toc262733384)

[5.1 Primary Efficacy Endpoint 10](#__RefHeading___Toc262733385)

[5.2 Secondary Efficacy Endpoints 10](#__RefHeading___Toc262733386)

[5.3 Safety Evaluations 10](#__RefHeading___Toc262733387)

[6 SUBJECT SELECTION 10](#__RefHeading___Toc262733389)

[6.1 Study Population 10](#__RefHeading___Toc262733390)

[6.2 Inclusion Criteria 10](#__RefHeading___Toc262733391)

[6.3 Exclusion Criteria 11](#__RefHeading___Toc262733392)

[7 Concurrent Medications 11](#__RefHeading___Toc262733393)

[7.1 Allowed 11](#__RefHeading___Toc262733394)

[7.2 Prohibited 11](#__RefHeading___Toc262733395)

[8 STUDY TREATMENTS 11](#__RefHeading___Toc262733396)

[8.1 Method of Assigning Subjects to Treatment Groups 11](#__RefHeading___Toc262733397)

[8.2 Blinding 11](#__RefHeading___Toc262733398)

[8.3 Test and Control Formulation 12](#__RefHeading___Toc262733399)

[8.4 Supply of Study Medication at the Site 13](#__RefHeading___Toc262733400)

[8.5 Study Medication Accountability 14](#__RefHeading___Toc262733401)

[9 STUDY PROCEDURES AND GUIDELINES 14](#__RefHeading___Toc262733403)

[9.1 Clinical Assessments 14](#__RefHeading___Toc262733404)

[10 EVALUATIONS BY VISIT 16](#__RefHeading___Toc262733408)

[10.1 Visit 1 (Day of surgery) 16](#__RefHeading___Toc262733409)

[10.2 Visit 2 (Postoperative day 1) 17](#__RefHeading___Toc262733410)

[10.3 Visit 3 (Postoperative week 1) 17](#__RefHeading___Toc262733411)

[10.6 Early Withdrawal Visit 18](#__RefHeading___Toc262733414)

[11 ADVERSE Experience REPORTING AND DOCUMENTATION 18](#__RefHeading___Toc262733415)

[11.1 Adverse Events 18](#__RefHeading___Toc262733416)

[11.2 Serious Adverse Experiences (SAE) 20](#__RefHeading___Toc262733417)

[11.4 Medical Monitoring 20](#__RefHeading___Toc262733419)

[11.5 Safety Management Plan](#__RefHeading___Toc262733420) 4

[12 DISCONTINUATION And Replacement of subjects 20](#__RefHeading___Toc262733421)

[12.1 Withdrawal of Subjects 20](#__RefHeading___Toc262733422)

[12.3 Replacement of Subjects 21](#__RefHeading___Toc262733423)

[13 PRotocol Violations 22](#__RefHeading___Toc262733424)

[14 STATISTICAL METHODS AND CONSIDERATIONS 22](#__RefHeading___Toc262733426)

[14.1 Data Sets Analyzed 22](#__RefHeading___Toc262733427)

[14.2 Demographic and Baseline Characteristics 22](#__RefHeading___Toc262733428)

[14.3 Analysis of Primary Endpoint 23](#__RefHeading___Toc262733429)

[14.4 Analysis of Secondary Endpoints 23](#__RefHeading___Toc262733430)

[14.5 Interim Analysis 23](#__RefHeading___Toc262733431)

[14.6 Sample Size and Randomization 23](#__RefHeading___Toc262733432)

[15 DATA COLLECTION, RETENTION AND MONITORING 23](#__RefHeading___Toc262733433)

[15.1 Data Collection Instruments 23](#__RefHeading___Toc262733434)

[15.2 Data Management Procedures 24](#__RefHeading___Toc262733435)

[15.3 Data Quality Control and Reporting 24](#__RefHeading___Toc262733436)

[15.4 Archival of Data 24](#__RefHeading___Toc262733437)

[15.5 Availability and Retention of Investigational Records 24](#__RefHeading___Toc262733438)

[15.6 Monitoring 25](#__RefHeading___Toc262733439)

[15.7 Subject Confidentiality 25](#__RefHeading___Toc262733440)

[16 ADMINISTRATIVE, ETHICAL, REGULATORY CONSIDERATIONS 25](#__RefHeading___Toc262733441)

[16.1 Protocol Amendments 25](#__RefHeading___Toc262733442)

[16.2 Institutional Review Boards and Independent Ethics Committees 25](#__RefHeading___Toc262733443)

[16.3 Informed Consent Form 26](#__RefHeading___Toc262733444)

[16.4 Publications 26](#__RefHeading___Toc262733445)

[16.5 Investigator Responsibilities 27](#__RefHeading___Toc262733446)

List of Abbreviations

| **AE** | adverse event |
| --- | --- |
| **BMI**  **CFR** | Body mass index  Code of Federal Regulations |
| **CRF** | case report form |
| **DMC** | Data Monitoring Committee |
| **DSMB** | Data Safety Monitoring Board |
| **FDA** | Food and Drug Administration |
| **GCP** | Good Clinical Practice |
| **HIPAA** | Health Insurance Portability and Accountability Act of 1996 |
| **ICF** | informed consent form |
| **ICH** | International Conference on Harmonisation |
| **IEC** | Independent Ethics Committee |
| **IRB** | Institutional Review Board |
| **PI** | Principal Investigator |
| **SAE**  **UCSF** | serious adverse experience  University of California, San Francisco |

Protocol Synopsis

| **TITLE** | **Optimal Location of Local Anesthetic Injection for Ultrasound-guided Interscalene Nerve Block** |
| --- | --- |
|  |  |
| **SPONSOR** | Pedram Aleshi, MD |
|  |  |
| **FUNDING ORGANIZATION** | Unfunded (miscellaneous departmental funding) |
| **NUMBER OF SITES** | 1 |
|  |  |
| **RATIONALE** | Patients undergoing shoulder surgery typically receive an ultrasound-guided interscalene nerve block preoperatively for postoperative pain control. Currently, the local anesthetic is injected either periplexus (in the space surrounding the interscalene brachial plexus in between the anterior and middle scalene muscles) or intraplexus (in between the nerve roots) depending on the preference of the anesthesiologist performing the block. However, the optimal location for injection of local anesthetic during ultrasound-guided interscalene nerve block is unknown.  We will prospectively randomize patients undergoing shoulder arthoscopy at the UCSF Orthopaedic Institute to receive either local anesthetic injection periplexus (circumferentially around the  interscalene plexus) or a single injection in between the nerve roots. |
|  |  |
| **STUDY DESIGN** | *This is a randomized, blinded study.* |
|  |  |
| **PRIMARY OBJECTIVE** | We hypothesize that local anesthetic injection intraplexus (meaning in between the nerve roots) rather than periplexus (circumferentially around plexus in between the scalene muscles) leads to faster onset of the interscalene block. The primary outcome variable will be time to onset of motor block. |
|  |  |
| **SECONDARY OBJECTIVES** | We hypothesize that the intraplexus approach will have a longer block duration, thus leading to reduced opioid consumption and greater  patient satisfaction postoperatively. We also hypothesize that the intraplexus technique will need to a faster procedure time, thus improving efficiency.  Secondary outcome variables that we will also investigate include: time to onset of sensory block, duration of nerve blockade, highest NRS pain score in the post anesthesia recovery unit and  postoperative day 1, block performance time, patient satisfaction score, perioperative opioid use, perioperative analgesic consumption, incidence of paresthesias, number of needle passes, incidence of postoperative nausea, vomiting, constipation, and any other complications. |
|  |  |
| **NUMBER OF SUBJECTS** | 208 |
|  |  |
| **SUBJECT SELECTION**  **CRITERIA** | Inclusion Criteria:  Age at least 18 years old, ASA I-II classification, scheduled for shoulder surgery  Exclusion Criteria:  Age younger than 18, non-English speaking, any contraindication for regional anesthesia, such as allergy to local anesthetics or opioids, coagulopathy or severe thrombocytopenia, infection at  puncture sites, pre-existing neuropathy in operative limb, need for postoperative nerve function monitoring, pulmonary disease or low baseline oxygen saturation, dementia, patient refusal, and high preoperative opioid requirements. |
|  |  |
| **TEST PRODUCT, DOSE, AND ROUTE OF ADMINISTRATION** | *25mL Ropivacaine 0.5% will be administered once via intraplexus brachial plexus block.* |
|  |  |
| **CONTROL PRODUCT, DOSE AND ROUTE OF ADMINISTRATION** | *25mL Ropivacaine 0.5% will be administered via extraplexus brachial plexus block.* |
|  |  |
| dURATION OF SUBJECT PARTICIPATION AND DURATION OF STUDY | Subjects will be on study for up to 15 days  **Screening:** up to 7 days  **Treatment:** 1 day  **Follow-up:** 7 days  The total duration of the study is expected to be 2 years. 24 months for subject recruitment and 24 months for final subject follow-up. |
| **E** |  |
| **CONCOMMITANT MEDICATIONS** | Allowed: midazolam, fentanyl, lidocaine, propofol, sevoflurane, desflurane, dexamethasone, ondansetron, prochlorperazine, metoclopramide, meperidine  Prohibited: Hydromorphone, ketorolac |
|  |  |
| **Efficacy Evaluations** |  |
| ***Primary endpoint*** | - Time to onset of motor block (loss of shoulder abduction) |
| ***Secondary endpoints*** | - Time to onset of sensory block - Duration of nerve blockade - Highest pain score in the post-anesthesia recovery - Highest pain score on post operative day 1 - Block performance time - Patient satisfaction score - Perioperative opioid use - Perioperative analgesic consumption - Number of needle passes - Block failure - Recovery room length of stay - Incidence of postoperative nausea, vomiting, constipation, and any other complications (hoarseness, shortness of breath, Horner’s syndrome, nerve injury, local anesthetic toxicity, infection at insertion site) - Incidence of paresthesias |
| **Safety Evaluations** | Incidence of adverse events will be recorded and monitored during the study. |
| **Planned Interim Analyses** | Serious adverse events will be monitored by an independent data monitoring committee on an ongoing basis throughout the study. |
|  |  |
| **STATISTICS**  **Primary Analysis Plan** | This study is designed as a prospective two treatment parallel-design study. The primary outcome is the time to block onset. Time-to-event data will be compared using the log-rank test. Assuming a true hazard  ratio of 0.65, power of 0.8 and alpha of 0.05, a sample size of 104 patients per group is needed. This is based on the assumption that the total number of events will be 172. |
| **Rationale for Number of Subjects** | The estimated sample size for a two-sample comparison of survivor functions for the primary outcome variable of time to onset of motor block using the Freedman method for log-rank test with a hazard ratio of 0.65, Power of 0.8 and alpha of 0.05, was a total of 208 patients for the two groups, assuming a total number of 172 events. |

# BACKGROUND

Interscalene nerve blocks are used routinely for postoperative pain control of patients undergoing shoulder surgery. There is considerable evidence that ultrasound guidance increases the efficacy and decreases the performance time of interscalene blocks compared to landmark or nerve stimulation techniques (1,2). However, the optimal location for local anesthetic injection during ultrasound-guided interscalene nerve blocks is not known.

## Overview of Clinical Studies

Recent clinical studies suggest that subfascial injection results in faster block onset and success compared to conventional extrafascial techniques (3). Missair et al. compared extrafascial versus subfascial injection of local anesthetic for popliteal sciatic nerve block in a recent prospective randomized study of sixty patients (4). They found that the placement of the needle tip beneath the fascial sheath of the sciatic nerve resulted in greater sensory blockade (63% in the extrafascial group vs. 90% in the subfascial

group). This study suggests that subfascial injection of local anesthetic significantly improves the efficacy of the nerve block. One study conducted by Spence et al. examined subfascial versus extrafascial injection of local anesthetic in ultrasound-guided interscalene nerve blocks (5). This prospective randomized study of 170 patients

found no difference between the two groups in block onset or block quality. However, the duration of block was significantly longer in the subfascial group. Unfortunately, this study had several problems. They changed their protocol after enrolling 15 patients such that subsequent patients received a different dose of the local anesthetic. Additionally, although they powered the study for 208 patients, they only based the primary outcome of onset of motor block on 155 patients. Multiple injections were allowed in the subfascial group. We hypothesize that a single injection of local anesthetic between the

nerve roots (subfascial) compared to the traditional approach of circumferential, multiple injections around the interscalene plexus (extrafascial) will result in faster onset of nerve blockade, longer duration of blockade and greater patient satisfaction.

**References**

1. Kapral et al. Ultrasonographic guidance improves the success rate of interscalene brachial plexus blockade. Reg Anesth Pain Med 2008; 33: 253-258.
2. Liu et al. A prospective, randomized, controlled trial comparing ultrasound versus nerve stimulator guidance for ambulatory shoulder surgery for postoperative neurological symptoms. Anesth Analg 2009; 109: 265-271.
3. Choquet O, et al. Where should the tip of the needle be located in ultrasound-guided peripheral nerve blocks? Curr Opin Anaesthesiol 2012 Oct; 25(5):596-602
4. Missair et al. A 3-Dimensional ultrasound study of local anesthetic spread during lateral popliteal nerve block. Reg Anes Pain Med. 2012 Dec; 37(6): 627-632.
5. Spence et al. Ultrasound-guided interscalene blocks: understanding where to inject the local anaesthetic. Anaesthesia. 2011 Jun; 66(6): 509-514.

# STUDY RATIONALE

Although both intraplexus and extraplexus approaches to the interscalene brachial plexus blocks are performed, one approach may have benefits over the other. Previous studies in popliteal nerve blocks have found that the intraplexus approach was superior in terms of faster onset.

## Risk / Benefit Assessment

There are very rare, but serious risks to peripheral nerve blockade, including infection at the puncture site, bleeding at the puncture site, local anesthetic toxicity, anaphylaxis, and nerve injury. With interscalene nerve blockade, there is also a risk of Horner's syndrome, hoarseness, and respiratory distress. The more common risk of peripheral nerve blockade, occurring in less than 10% of blocks at the Orthopaedic Institute, is failure of the nerve block to provide adequate analgesia in the corresponding anatomical site. This can be easily treated postoperatively by either administration of IV pain medications or a postoperative nerve block. Patients undergoing nerve blocks are advised to protect the operative limb in a sling until numbness from the block has resolved. The numbness resulting from the nerve block is a common and desired side effect. Of note, these risks are not related to patient participation in the study, but rather are the risks of peripheral nerve blockade which patients would receive even if they were not part of the study.

Specific study risks include a potential loss of privacy for patients that are involved in the study. There is a potentially higher risk of transient paresthesia in the group receiving intraplexus interscalene nerve blocks, which we will be recording. There is also a risk with randomization that the patients will be assigned to a treatment program by chance and the treatment they receive may prove to be less effective or to have more side effects than the other study treatment or other available treatments.

**Steps taken to minimize risk to subjects**:

Nerve blocks are performed under sterile conditions with topical application of chlorhexidine to minimize risk of infection. Ultrasound guidance is used to ensure avoidance of needle-to-nerve contact and intravascular injection of local anesthetic. Intravascular injection is prevented by frequent aspiration during local anesthetic injection. In addition to the use of ultrasound real-time guidance, intraneural injection is prevented by performing the block on awake patients with minimal sedation such that they are able to report any pain or paresthesia on injection. Intraneural injection is

also identified by increased resistance on injection. Visualization of local anesthetic spread around the desired nerve is highly predictive for a successful block.

In this study, the preoperative and postoperative nerve blocks are performed in the preoperative holding area and post anesthesia recovery area, respectively. In both locations, the blocks are performed under continuous non-invasive blood pressure and pulse oximetry monitoring. Administration of sedation is minimized so that patients can report paresthesias, should they occur. Emergency medications and equipment is readily available.

The subjects in this study will have a more thorough post-anesthesia follow-up for pain and other side effects. Understanding the optimal location for local anesthetic deposition for peripheral nerve blocks which result in greater patient comfort following ambulatory shoulder arthroscopy is of benefit to society. Complications from single-shot peripheral nerve blockade overall and at our institution, have been extraordinarily rare. Interscalene nerve blocks are commonly employed in anesthetic practice.

# STUDY OBJECTIVES

## Primary Objective

The primary objective is to assess the time to motor onset of interscalene brachial plexus block, as measured by the loss of shoulder abduction, for two approaches to interscalene block (intraplexus and extraplexus)

## Secondary Objectives

- Time to onset of sensory block
- Duration of nerve blockade
- Highest pain score in the post-anesthesia recovery
- Highest pain score on postoperative day 1 and at postoperative week 1
- Block performance time
- Patient satisfaction score
- Perioperative opioid use
- Perioperative analgesic consumption
- Number of needle passes
- Block failure
- Recovery room length of stay
- Incidence of postoperative nausea, vomiting, constipation, and any other complications (hoarseness, shortness of breath, Horner’s syndrome, nerve injury, local anesthetic toxicity, infection at insertion site)
- Incidence of paresthesias

# STUDY DESIGN

## Study Overview

This is a single center, blinded, randomized trial. 208 of subjects are planned. Each subject will be administered a single injection of ropivacaine 0.5% either intraplexus or extraplexus prior to their shoulder surgery. Subjects will be assigned to the treatment group in random order. Evaluations will be taken in one-minute intervals after placement of the block until loss of motor or sensation is observed. Subjects will undergo a standardized general anesthetic. Sensory exam will be reevaluated in the recovery room. Patients will be contacted via telephone on post-operative day 1. Data from their 1 week postoperative visit will be recorded.

Screening data will be reviewed to determine subject eligibility. Subjects who meet all inclusion criteria and none of the exclusion criteria will be entered into the study.

The following treatment regimens will be used:

Experimental treatment –interscalene injection performed intraplexus (between C5-C6 nerve roots)

Comparator – interscalene injection performed extraplexus (anterior and posterior to brachial plexus in interscalene groove)

Total duration of subject participation will be two weeks. Total duration of the study is expected to be 24 months.

# Criteria for evaluation

## Primary Efficacy Endpoint

Time to loss of shoulder abduction. Patient will be sitting upright and instructed to abduct arm every 1 minute until they are no longer able to.

## Secondary Efficacy Endpoints

- Time to onset of sensory block – evaluated by detection of decreased sharpness of contralateral side. This will be evaluated for supraclavicular, axillary, musculocutaneous, median and ulnar nerve distributions.
- Duration of nerve blockade (time from block completion to time patient reports the block started to wear off and no longer providing pain relief)
- Highest pain score in the post-anesthesia recovery unit (using numerical rating scale from 0-10 pain, 0 is no pain, 10 is worst imaginable pain)
- Highest pain score on postoperative day 1 (using numerical rating scale from 0-10 pain, 0 is no pain, 10 is worst imaginable pain), at rest and with movement
- Block performance time - time from block needle insertion to the time that all of the local anesthetic was injected
- Patient satisfaction score – scale from 0-10. Zero is unsatisfied. 5 is neutral and 10 is extremely satisfied.
- Perioperative opioid use – total opioids used preoperative, intraoperative, and postoperatively will be recorded
- Perioperative analgesic consumption – acetaminophen, ibuprofen, ketorolac usage perioperatively
- Number of needle passes - number of times the needle was re-advanced after being withdrawn during block placement
- Block failure - lack of sensory exam consistent with interscalene block in the recovery room or need for repeat interscalene block in recovery room
- Recovery room length of stay (time from entering recovery room to time that discharge criteria met)
- Highest pain score at one week postoperative week visit (0-10 with zero being no pain and 10 worst imaginable pain)

## Safety Evaluations

Incidence of:

- - Postoperative nausea and vomiting
  - Constipation
  - Hoarseness
  - shortness of breath
  - Horner’s syndrome
  - local anesthetic toxicity – any metallic taste in the mouth, tinnitus, altered mental status, hemodynamic instability related to block placement
  - infection at insertion site
  - paresthesias
  - nerve injury (defined as persistent nerve deficit)

# SUBJECT SELECTION

## Study Population

Subjects who are scheduled for an ambulatory arthroscopic shoulder surgery who meet the inclusion and exclusion criteria will be eligible for participation in this study.

## Inclusion Criteria

1. Male or female ≥18years of age at time of surgery.
2. ASA I-II classification, scheduled for ambulatory shoulder surgery at the UCSF Orthopaedic Institute.
3. Written informed consent (and assent when applicable) obtained from subject or subject’s legal representative and ability for subject to comply with the requirements of the study.

## Exclusion Criteria

1. Pregnant during participation in the study.
2. Non-English speaking
3. Any contraindication for regional anesthesia, such as an allergy to local anesthetics or opioids, coagulopathy or severe thrombocytopenia, infection at puncture sites, pre-existing neuropathy in operative limb, need for postoperative nerve function monitoring
4. History of pulmonary disease or low baseline oxygen saturation
5. History of dementia
6. High preoperative opioid requirements

# Concurrent Medications

All subjects should be maintained on the same medications throughout the entire study period, as medically feasible, with no introduction of new chronic therapies.

## Allowed Medications and Treatments

Standard therapy for anesthesia for shoulder surgery is allowed except for treatments noted in the exclusion criteria described above and as noted in the prohibited medications section below. 

Prohibited Medications and Treatments

The following medications are prohibited during the study and administration will be considered a protocol violation.

- Intraoperative ketorolac or hydromorphone

# STUDY TREATMENTS

## Method of Assigning Subjects to Treatment Groups

Up to 208 eligible patients will be randomly assigned to intraplexus or extraplexus treatment groups in a 1:1 ratio using an Excel computer-generated randomization scheme developed by the study data management provider. The allocated group will be written on a paper in a sealed, opaque envelope that will be provided only to the anesthesia provider performing the block.

## Blinding

Due to the objectives of the study, the identity of test and control treatments will not be known to investigators, research staff, or patients. The following study procedures will be in place to ensure double-blind administration of study treatments.

Access to the randomization code will be strictly controlled. The study blind will be broken on completion of the clinical study and after the study database has been locked. Investigators will not be made aware of their subjects’ treatment assignments. All providers, who administer pain medications to the patient, will be blinded to their treatment assignment.

During the study, the blind may be broken **only** in emergencies when knowledge of the patient’s treatment group is necessary for further patient management. When possible, the Investigator should discuss the emergency with the Principal Investigator prior to unblinding.

## Formulation of Test and Control Products

Both test and control treatment groups will receive 25mL of Ropivacaine 0.5%, a local anesthetic that is approved for peripheral nerve block use. Ropivacaine is a clear colored solution that requires no reconstitution. There is no placebo comparator in this study as it would be unethical given the analgesic benefits of interscalene block for shoulder surgery. As the drug is the same for each group, it is not necessary for it to be blinded.

## Supply of Study Drug at the Site

As the study drug is a commonly used medication for peripheral nerve block, it is readily available at the study site.

### Dosage/Dosage Regimen

25mL of ropivacaine 0.5% given for interscalene block once. No adjustments for age. This dose must not exceed a max dose of 3.5mg/kg of ropivacaine.

Dispensing

The anesthesia provider performing the block will prepare and dispense the drug.

### Administration Instructions

**For the placement of the interscalene block, the patient may receive any of the following medications:**

- Midazolam: 0-4mg IV
- Fentanyl: 0-100mcg IV
- Ropivacaine 0.5%: 25mL for interscalene block

**For the induction of general anesthesia:**

- Lidocaine: 0-100mg IV
- Propofol: as much as necessary for IV induction
- Fentanyl: 0-50mcg IV for LMA insertion

**For the maintenance of general anesthesia:**

- Sevoflurane or desflurane per anesthesia provider.
- Propofol gtt between 25-100mcg/kg/min per anesthesia provider.

**For IntraOperative Analgesia:**

- Fentanyl: as needed at anesthesia provider's discretion.
- No Ketorolac or Hydromorphone (IntraOp).

**For IntraOperative PONV prophylaxis:**

- Dexamethasone: 4mg IV after induction
- Ondansetron: 4mg IV prior to emergence

**For recovery room orders:**

- Fentanyl: 0-350mcg PRN pain
- Ketorolac 30mg x1 in PACU only
- No hydromorphone orders until evaluated by study personnel
- Ondansetron: 4mg IV (2nd dose)
- Prochlorperazine 10mg IV x1 dose
- Metoclopramide 10mg IV x1 dose
- Meperidine up to 50mg for shivering

## Supply of Study Drug at the Site

The study drug is a commonly used local anesthetic that is readily available at the study site.

### Storage

Study drug should be stored by the study site at controlled room temperature, 15 to 30ºC (59 to 86ºF). If the temperature of study drug storage in the clinic/pharmacy exceeds or falls below this range, this should be reported to the Principal Investigator or designee and captured as a deviation. Subjects will be instructed to store the medication in original packaging (foil pouch and protected from light) at room temperature according to the instructions outlined on the Drug Administration Instructions.

## Study Drug Accountability

An accurate and current accounting of the dispensing and return of study drug for each subject will be maintained on an ongoing basis by a member of the study site staff. The amount of study drug dispensed will be recorded in the Medication Administration Record.

# STUDY PROCEDURES AND GUIDELINES

A Schedule of Events representing the required testing procedures to be performed for the duration of the study is diagrammed in Appendix 1.

Prior to conducting any study-related activities, written informed consent and the Health Insurance Portability and Accountability Act (HIPAA) authorization must be signed and dated by the subject. If appropriate, assent must also be obtained prior to conducting any study-related activities.

## Clinical Assessments

### Concomitant Medications

All concomitant medication and concurrent therapies will be documented at screening and on study day 1 (day of surgery), postoperative day 1, and at early termination when applicable. Dose, route, unit frequency of administration, and indication for administration and dates of medication will be captured.

### Demographics

Demographic information (date of birth, gender, race) will be recorded at Screening.

### Medical History

Relevant medical history, including history of current disease, other pertinent respiratory history, and information regarding underlying diseases will be recorded at Screening.

### Physical Examination

A complete physical examination will be performed by either the investigator or a subinvestigator who is a physician at Visit #1. Qualified staff (MD, NP, RN, and PA) may complete the abbreviated physical exam at all other visits. New abnormal physical exam findings must be documented and will be followed by a physician or other qualified staff at the next scheduled visit.

### Vital Signs

Body temperature, blood pressure, pulse and respirations will be performed after resting for 5 minutes on Visit day 1.

### Oximetry

Oximetry will be measured on room air with the subject at rest at Visit 1.

### Other Clinical Procedures

**Interscalene nerve block:**

Patients at the Orthopaedic Institute undergoing shoulder arthoscopy typically receive a preoperative ultrasound-guided interscalene nerve block. Patients in the control group will have local anesthetic injected circumferentially around the interscalene brachial plexus. Patients in the experimental group will have a single injection of local anesthetic placed inbetween the C5- C6 nerve roots. Light sedation is usually administered via the patient's IV during performance of the block. These blocks are performed in the preoperative holding area, with the patients receiving continuous non-invasive blood pressure and pulse oximetry monitoring until being transported into the operating room.

An study investigator (not involved in block placement) will assess motor and sensory block until the onset of block or until the patient is taken to the operating room. From this point forward, patients in both groups are treated identically. All patients will subsequently undergo a general anesthestic.

Postoperatively, if the patient has poor pain control with intravenous opioids, they will be evaluated by an anesthesiologist. A nerve block may be repeated postoperatively if deemed necessary. This nerve block occurs in the post anesthesia recovery unit under continuous noninvasive blood pressure and pulse oximetry monitoring. Patients requiring postoperative nerve blocks will continue to participate in the study, and the number of patients requiring such blocks will be tabulated and analyzed as one of our outcome variables. All patients will be asked to rate their pain and will be provided a pain diary while in the recovery unit.

On postoperative day 1, an anesthesiologist will call the patient and review the questions on the pain diary and solicit responses from the patient. The patient will not be asked to return the pain diary -- it is meant to serve as a guide for the patient as to what questions they will be asked during these brief, approximately 5 minute, phone calls. If the patient's block has not yet resolved by the time of the follow-up phone call on postoperative day 1, then the patient will be called daily until resolution of their block, typically by postoperative day 2.

The patients will be seen by the orthopedic surgery team on 1 week postoperatively and will be assessed for any adverse events as well as their highest pain score.

### Adverse Events

Information regarding occurrence of adverse events will be captured throughout the study. Duration (start and stop dates), severity/grade, outcome, treatment and relation to study drug will be recorded on the case report form (CRF).

### Pregnancy Test

A urine pregnancy test will be obtained from female subjects who are of childbearing age prior to their participation in the study.

# EVALUATIONS BY VISIT

## Visit 1 (Day of surgery)

1. Review the study with the subject and obtain written informed consent and HIPAA authorization and assent, if appropriate.
2. Assign the subject a unique screening number.
3. Record demographics data.
4. Record medical history.
5. Record concomitant medications.
6. Perform a complete physical examination.
7. Perform and record vital signs.
8. Perform and record oximetry.
9. Perform and record results of blood pressure testing.
10. Collect urine for pregnancy test if applicable.
11. Randomize subject.
12. Perform interscalene block.
13. Assess subject for onset of motor and sensory blockade prior to entering operating room.
14. Assess subject for sensory blockade in recovery room.
15. Assess subject for any adverse events.
16. Record medications used intraoperatively and postoperatively.
17. Initiate subject diary.

## Visit 2 (postoperative day 1)

1. Record any Adverse Experiences and/or Review subject diary for adverse experiences and dosing compliance.
2. Concomitant medications review.
3. Record pain medications.
4. Record patient satisfaction, incidence of postoperative nausea and vomiting.
5. List all additional procedures.

## Visit 3 (postoperative week 1)

1. Record any Adverse Experiences and/or Review subject diary for adverse experiences.
2. Record changes to concomitant medications.
3. Perform abbreviated physical examination.
4. Perform and record vital signs.
5. Assess pain score.

## Early Withdrawal Visit

1. Record any Adverse Experiences and/or Review subject diary for adverse experiences and exclusionary medication use.
2. Record changes to concomitant medications.
3. Perform and record vital signs and physical exam.

# ADVERSE Experience REPORTING AND DOCUMENTATION

## Adverse Events

An adverse event (AE) is any untoward medical occurrence in a clinical investigation of a patient administered a pharmaceutical product and that does not necessarily have a causal relationship with the treatment. An AE is therefore any unfavorable and unintended sign (including an abnormal laboratory finding), symptom or disease temporally associated with the administration of an investigational product, whether or not related to that investigational product. An unexpected AE is one of a type not identified in nature, severity, or frequency in the current Investigator’s Brochure or of greater severity or frequency than expected based on the information in the Investigator’s Brochure.

The Investigator will probe, via discussion with the subject, for the occurrence of AEs during each subject visit and record the information in the site’s source documents. Adverse events will be recorded in the patient CRF. Adverse events will be described by duration (start and stop dates and times), severity, outcome, treatment and relation to study drug, or if unrelated, the cause.

**AE Severity**

The National Cancer Institute’s Common Terminology Criteria for Adverse Events (CTCAE) Version 3.0 should be used to assess and grade AE severity, including laboratory abnormalities judged to be clinically significant. The modified criteria can be found in the study manual. If the experience is not covered in the modified criteria, the guidelines shown in Table 1 below should be used to grade severity. It should be pointed out that the term “severe” is a measure of intensity and that a severe AE is not necessarily serious.

Table 1. AE Severity Grading

| **Severity (Toxicity Grade)** | **Description** |
| --- | --- |
| Mild (1) | Transient or mild discomfort; no limitation in activity; no medical intervention or therapy required. The subject may be aware of the sign or symptom but tolerates it reasonably well. |
| Moderate (2) | Mild to moderate limitation in activity, no or minimal medical intervention/therapy required. |
| Severe (3) | Marked limitation in activity, medical intervention/therapy required, hospitalizations possible. |
| Life-threatening (4) | The subject is at risk of death due to the adverse experience as it occurred. This does not refer to an experience that hypothetically might have caused death if it were more severe. |

**AE Relationship to Study Drug**

The relationship of an AE to the study drug should be assessed using the following the guidelines in Table 2.

Table 2. AE Relationship to Study Drug

| **Relationship to Drug** | **Comment** |
| --- | --- |
| Definitely | Previously known toxicity of agent; or an event that follows a reasonable temporal sequence from administration of the drug; that follows a known or expected response pattern to the suspected drug; that is confirmed by stopping or reducing the dosage of the drug; and that is not explained by any other reasonable hypothesis. |
| Probably | An event that follows a reasonable temporal sequence from administration of the drug; that follows a known or expected response pattern to the suspected drug; that is confirmed by stopping or reducing the dosage of the drug; and that is unlikely to be explained by the known characteristics of the subject’s clinical state or by other interventions. |
| Possibly | An event that follows a reasonable temporal sequence from administration of the drug; that follows a known or expected response pattern to that suspected drug; but that could readily have been produced by a number of other factors. |
| Unrelated | An event that can be determined with certainty to have no relationship to the study drug. |

## Serious Adverse Experiences (SAE)

An SAE is defined as any AE occurring at any dose that results in any of the following outcomes:

- death
- a life-threatening adverse experience
- inpatient hospitalization or prolongation of existing hospitalization
- a persistent or significant disability/incapacity
- a congenital anomaly/birth defect

Other important medical events may also be considered an SAE when, based on appropriate medical judgment, they jeopardize the subject or require intervention to prevent one of the outcomes listed.

### Serious Adverse Experience Reporting

Study sites will document all SAEs that occur (whether or not related to study drug) per [UCSF CHR Guidelines](http://www.research.ucsf.edu/chr/Guide/Adverse_Events_Guidelines.asp#2). The collection period for all SAEs will begin after informed consent is obtained and end after procedures for the final study visit have been completed.

In accordance with the standard operating procedures and policies of the local Institutional Review Board (IRB)/Independent Ethics Committee (IEC), the site investigator will report SAEs to the IRB/IEC.

## Medical Monitoring

Monica Harbell should be contacted directly at these numbers to report medical concerns or questions regarding safety.

Phone: (415) 476-0936

Pager: (415) 443-3085

# DISCONTINUATION And Replacement of subjects

## Early Discontinuation of Study Drug

A subject may be discontinued from study treatment at any time if the subject, the investigator, or the Sponsor feels that it is not in the subject’s best interest to continue. The following is a list of possible reasons for study treatment discontinuation:

Subject withdrawal of consent (or assent)

Subject is not compliant with study procedures

Adverse event that in the opinion of the investigator would be in the best interest of the subject to discontinue study treatment

Protocol violation requiring discontinuation of study treatment

Lost to follow-up

Sponsor request for early termination of study

Positive pregnancy test (females)

If a subject is withdrawn from treatment due to an adverse event, the subject will be followed and treated by the Investigator until the abnormal parameter or symptom has resolved or stabilized.

All subjects who discontinue study treatment should come in for an early discontinuation visit as soon as possible and then should be encouraged to complete all remaining scheduled visits and procedures.

All subjects are free to withdraw from participation at any time, for any reason, specified or unspecified, and without prejudice.

Reasonable attempts will be made by the investigator to provide a reason for subject withdrawals. The reason for the subject’s withdrawal from the study will be specified in the subject’s source documents Refer to Section 10 for early termination procedures.

## Withdrawal of Subjects from the Study

A subject may be withdrawn from the study at any time if the subject, the investigator, or the Sponsor feels that it is not in the subject’s best interest to continue.

All subjects are free to withdraw from participation at any time, for any reason, specified or unspecified, and without prejudice.

Reasonable attempts will be made by the investigator to provide a reason for subject withdrawals.  The reason for the subject’s withdrawal from the study will be specified in the subject’s source documents.  As noted above, subjects who discontinue study treatment early (i.e., they withdraw prior to Visit 2) should have an early discontinuation visit. Refer to Section 10 for early termination procedures.  Subjects who withdraw after Visit 2 but prior to Visit 3 should be encouraged to come in for a final visit (and the procedures to be followed would include those for their next scheduled visit).

## Replacement of Subjects

Subjects who withdraw from the study treatment will not be replaced.

Subjects who withdraw from the study will not be replaced.

# PRotocol Violations

A protocol violation occurs when the subject, investigator, or Sponsor fails to adhere to significant protocol requirements affecting the inclusion, exclusion, subject safety and primary endpoint criteria. Protocol violations for this study include, but are not limited to, the following:

Failure to meet inclusion/exclusion criteria

Use of a prohibited concomitant medication

Failure to comply with Good Clinical Practice (GCP) guidelines will also result in a protocol violation. The Sponsor will determine if a protocol violation will result in withdrawal of a subject.

When a protocol violation occurs, it will be discussed with the investigator and a Protocol Violation Form detailing the violation will be generated. This form will be signed by a Sponsor representative and the Investigator. A copy of the form will be filed in the site’s regulatory binder and in the Sponsor’s files.

# STATISTICAL METHODS AND CONSIDERATIONS

Prior to the analysis of the final study data, a detailed Statistical Analysis Plan (SAP) will be written describing all analyses that will be performed. The SAP will contain any modifications to the analysis plan described below.

## Data Sets Analyzed

All eligible patients who are randomized into the study and receive an interscalene block will be included in the safety analysis.

## Demographic and Baseline Characteristics

The following demographic variables at screening will be summarized by intervention groups: gender, age, height, weight, BMI. The following day of surgery characteristics will be recorded on the day of surgery: surgeon performing surgery, duration of surgery, conversion to open surgery.

## Analysis of Primary Endpoint

This study is designed as a prospective two treatment parallel-design study. The primary outcome is the time to block onset. Time-to-event data will be compared using the log-rank test.

## Analysis of Secondary Endpoints

Categorical data will be analyzed using Chi squared analysis or Fischer's exact test, depending on sample size. Comparison of means will be performed using the independent sample t-test or the Mann-Whitney U test, depending on distribution. A p-value of 0.05 or smaller will be considered statistically significant.

Safety and tolerability data will be summarized by treatment group. Adverse event rates will be coded by body system and MedDra classification term.  Adverse events will be tabulated by treatment group and will include the number of patients for whom the event occurred, the rate of occurrence, and the severity and relationship to study drug.

## Interim Analysis

This study does not entail any sequential or adaptive trial designs and as such, does not require an interim analysis.

## Sample Size and Randomization

The estimated sample size for a two-sample comparison of survivor functions for the primary outcome variable of time to onset of motor block using the Freedman method for log-rank test with a hazard ratio of 0.65, Power of 0.8 and alpha of 0.05, was a total of 208 patients for the two groups, assuming a total number of 172 events.

DATA COLLECTION, RETENTION AND MONITORING

## Data Collection Instruments

The Investigator will prepare and maintain adequate and accurate source documents designed to record all observations and other pertinent data for each subject treated with the study drug.

Study personnel at each site will enter data from source documents corresponding to a subject’s visit into the protocol-specific paper Case Report Form (CRF) when the information corresponding to that visit is available. Subjects will not be identified by name in the study database or on any study documents to be collected by the Sponsor (or designee), but will be identified by a subject number. If a correction is made on a CRF, the study staff member will line through the incorrect data, write in the correct data and initial and date the change.

The Investigator is responsible for all information collected on subjects enrolled in this study. All data collected during the course of this study must be reviewed and verified for completeness and accuracy by the Investigator. A copy of the CRF will remain at the Investigator’s site at the completion of the study.

## Data Management Procedures

The data will be entered into a validated database. The Data Management group will be responsible for data processing, in accordance with procedural documentation. Database lock will occur once quality assurance procedures have been completed.

All procedures for the handling and analysis of data will be conducted using good computing practices meeting FDA guidelines for the handling and analysis of data for clinical trials.

## Data Quality Control and Reporting

After data have been entered into the study database, a system of computerized data validation checks will be implemented and applied to the database on a regular basis. The study database will be updated in accordance with the resolved queries. All changes to the study database will be documented.

## Archival of Data

The database is safeguarded against unauthorized access by established security procedures; appropriate backup copies of the database and related software files will be maintained.  Databases are backed up by the database administrator in conjunction with any updates or changes to the database.

At critical junctures of the protocol (e.g., production of interim reports and final reports), data for analysis is locked and cleaned per established procedures.

## Availability and Retention of Investigational Records

The Investigator must make study data accessible to the monitor, other authorized representatives of the Sponsor (or designee), IRB/IEC, and Regulatory Agency (e.g., FDA) inspectors upon request. A file for each subject must be maintained that includes the signed Informed Consent, HIPAA Authorization and Assent Form and copies of all source documentation related to that subject. The Investigator must ensure the reliability and availability of source documents from which the information on the CRF was derived.

All study documents (patient files, signed informed consent forms, copies of CRFs, Study File Notebook, etc.) must be kept secured for a period of two years following marketing of the investigational product or for two years after centers have been notified that the IND has been discontinued. There may be other circumstances for which the Sponsor is required to maintain study records and, therefore, the Sponsor should be contacted prior to removing study records for any reason.

## Monitoring

Monitoring visits will be conducted by representatives of the Sponsor according to the U.S. CFR Title 21 Parts 50, 56, and 312 and ICH Guidelines for GCP (E6). By signing this protocol, the Investigator grants permission to the Sponsor (or designee), and appropriate regulatory authorities to conduct on-site monitoring and/or auditing of all appropriate study documentation.

## Subject Confidentiality

In order to maintain subject confidentiality, only a subject number will identify all study subjects on CRFs and other documentation submitted to the Sponsor. Additional subject confidentiality issues (if applicable) are covered in the Clinical Study Agreement.

# ADMINISTRATIVE, ETHICAL, REGULATORY CONSIDERATIONS

The study will be conducted according to the Declaration of Helsinki, Protection of Human Volunteers (21 CFR 50), Institutional Review Boards (21 CFR 56), and Obligations of Clinical Investigators (21 CFR 312).

To maintain confidentiality, all laboratory specimens, evaluation forms, reports and other records will be identified by a coded number and initials only. All study records will be kept in a locked file cabinet and code sheets linking a patient’s name to a patient identification number will be stored separately in another locked file cabinet. Clinical information will not be released without written permission of the subject, except as necessary for monitoring by the FDA. The Investigator must also comply with all applicable privacy regulations (e.g., Health Insurance Portability and Accountability Act of 1996, EU Data Protection Directive 95/46/EC).

## Protocol Amendments

Any amendment to the protocol will be written by the principal investigator. Protocol amendments cannot be implemented without prior written IRB/IEC approval except as necessary to eliminate immediate safety hazards to patients. A protocol amendment intended to eliminate an apparent immediate hazard to patients may be implemented immediately, provided the IRBs are notified within five working days.

## Institutional Review Boards and Independent Ethics Committees

The protocol and consent form will be reviewed and approved by the IRB/IEC of each participating center prior to study initiation. Serious adverse experiences regardless of causality will be reported to the IRB/IEC in accordance with the standard operating procedures and policies of the IRB/IEC, and the Investigator will keep the IRB/IEC informed as to the progress of the study. The Investigator will obtain assurance of IRB compliance with regulations.

Any documents that the IRB may need to fulfill its responsibilities (such as protocol, protocol amendments, Investigator’s Brochure, consent forms, information concerning patient recruitment, payment or compensation procedures, or other pertinent information) will be submitted to the IRB. The IRBs written unconditional approval of the study protocol and the informed consent form will be in the possession of the Investigator before the study is initiated.

Protocol and/or informed consent modifications or changes may not be initiated without prior written IRB approval except when necessary to eliminate immediate hazards to the patients or when the change(s) involves only logistical or administrative aspects of the study. Such modifications will be submitted to the IRB/IEC and written verification that the modification was submitted and subsequently approved should be obtained.

The IRB must be informed of revisions to other documents originally submitted for review; serious and/or unexpected adverse experiences occurring during the study in accordance with the standard operating procedures and policies of the IRB; new information that may affect adversely the safety of the patients of the conduct of the study; an annual update and/or request for re-approval; and when the study has been completed.

## Informed Consent Form

Informed consent will be obtained in accordance with the Declaration of Helsinki, ICH GCP, US Code of Federal Regulations for Protection of Human Subjects (21 CFR 50.25[a,b], CFR 50.27, and CFR Part 56, Subpart A), the Health Insurance Portability and Accountability Act (HIPAA, if applicable), and local regulations.

The Investigator will prepare the informed consent form, assent and HIPAA authorization and provide the documents to the Sponsor or designee for approval prior to submission to the IRB/IEC. The consent form generated by the Investigator must be acceptable to the Sponsor and be approved by the IRB/IEC. The written consent document will embody the elements of informed consent as described in the International Conference on Harmonisation and will also comply with local regulations. The Investigator will send an IRB/IEC-approved copy of the Informed Consent Form to the Sponsor (or designee) for the study file.

A properly executed, written, informed consent will be obtained from each subject prior to entering the subject into the trial. Information should be given in both oral and written form and subjects must be given ample opportunity to inquire about details of the study. If appropriate and required by the local IRB/IEC, assent from the subject will also be obtained. If a subject is unable to sign the informed consent form (ICF) and the HIPAA authorization, a legal representative may sign for the subject. A copy of the signed consent form (and assent) will be given to the subject and the original will be maintained with the subject’s records.

## Publications

The preparation and submittal for publication of manuscripts containing the study results shall be in accordance with a process determined by mutual written agreement among the study Sponsor and participating institutions. The publication or presentation of any study results shall comply with all applicable privacy laws, including, but not limited to, the Health Insurance Portability and Accountability Act of 1996.

## Investigator Responsibilities

By signing the Agreement of Investigator form, the Investigator agrees to:

1. Conduct the study in accordance with the protocol and only make changes after notifying the Sponsor (or designee), except when to protect the safety, rights or welfare of subjects.
2. Personally conduct or supervise the study (or investigation).
3. Ensure that the requirements relating to obtaining informed consent and IRB review and approval meet federal guidelines, as stated in § 21 CFR, parts 50 and 56.
4. Report to the Sponsor or designee any AEs that occur in the course of the study, in accordance with §21 CFR 312.64.
5. Ensure that all associates, colleagues and employees assisting in the conduct of the study are informed about their obligations in meeting the above commitments.
6. Maintain adequate and accurate records in accordance with §21 CFR 312.62 and to make those records available for inspection with the Sponsor (or designee).
7. Ensure that an IRB that complies with the requirements of §21 CFR part 56 will be responsible for initial and continuing review and approval of the clinical study.
8. Promptly report to the IRB and the Sponsor (or designee) all changes in the research activity and all unanticipated problems involving risks to subjects or others (to include amendments and IND safety reports).
9. Seek IRB approval before any changes are made in the research study, except when necessary to eliminate hazards to the patients/subjects.
10. Comply with all other requirements regarding the obligations of clinical investigators and all other pertinent requirements listed in § 21 CFR part 312.

APPENDIX 1. Example of Schedule of Study Visits

|  | **Visit 1 (Day of surgery) ^a^** | **Visit 2 (Postoperative day 1)^a^** | **Visit 3 (1 week postoperative)^a^** |
| --- | --- | --- | --- |
| Informed Consent | **x** |  |  |
| Medical History | **x** |  |  |
| Complete Physical Exam | **x** |  |  |
| Abbreviated Physical Exam |  |  | **x** |
| Height | **x** |  |  |
| Weight | **x** |  |  |
| Vital Signs | **x** |  |  |
| Oximetry | **x** |  |  |
| Pregnancy Test (Urine) | **x** |  |  |
| Randomization | **x** |  |  |
| Administration of Study Drug | **X** |  |  |
| Initiate Subject Diary | **x** |  |  |
| Subject Diary Review |  | **X** |  |
| Motor and sensory exam | **X** |  |  |
| Pain score assessment | **X** | **X** | **X** |
| Opioid consumption | **X** | **X** |  |
| Postoperative telephone questions (block duration, pain, satisfaction) |  | **x** |  |
| Adverse Experiences | **X** | **X** | **X** |

^a^ ±2
