# Supplementary material for: Extraplexus versus intraplexus ultrasound-guided interscalene brachial plexus block for ambulatory arthroscopic shoulder surgery: A randomized controlled trial
Source: PLoS One. 2021 Feb 18;16(2):e0246792. doi: 10.1371/journal.pone.0246792 (PMC7891753; doi:10.1371/journal.pone.0246792)
Supplement: Attachment — Submitted filename: ReviewerforPLOS2020.docx [file pone.0246792.s007.docx]

**Reviewer’s comments to the authors**

Thank you for asking me to review the work ‘Extraplexus versus Intraplexus Ultrasound-Guided Interscalene Brachial Plexus Block for Ambulatory Arthroscopic Shoulder Surgery: a

randomized controlled trial’ by Harbell and colleagues.

The paper is written well, and I congratulate the authors for their work.

However, I have a few concerns, some of which can be addressed while others cannot.

1. This is an old study. As the authors mention, it was registered in 2012 and the results were presented in 2016.
2. The authors have used a large volume of local anesthetic for the block. They do give a valid reason.
3. Sample size
   a. Sample size was not adjusted for dropouts. This always leads to problems when there is missing data, postoperatively. In this study, the sample size for the primary objective was 104 per group. In statistical sense, authors did not reach the sample size that they were supposed to. They only had 103 patients in one group and though the difference is only 1, mathematically and statistically, they did not attain the calculated sample. This problem would not have happened if they had increased their sample by 10% (or any number, for that matter).
   b. The authors should explain why there was unequal distribution of patients across the 2 groups resulting in inadequate sample in the intraplexus group (105 vs 103).
   c. The primary outcome was time to block and the sample size was calculated for this outcome only. Paresthesia is the only secondary outcome significant finding (other than the primary outcome). However, it was a secondary outcome and the sample was not calculated for this outcome. This should be mentioned in the limitations.
4. In the extraplexus group, one patient was lost to followup but the n in the tables remains 105. Similarly, there were 2 patients in the intraplexus group, but the n remains 103.
5. Unblinding is a very big limitation in a double-blind trial, however small the percentage may be. It would be best to use single blind and remove the sections explaining 10% blinding and lack of staffing. Lack of staffing is a local matter that everyone faces but never acknowledges in scientific studies. For most ethics review boards, one has to ascertain that they have enough staff, resources and funding to be able to do the study. Therefore, one cannot (should not) say that during the study, they did not have enough staff.
6. Paresthesia is an important secondary outcome of this study. The authors should spend a few lines describing what they considered paresthesia in the context of this work.
7. Conclusions: The conclusions focus on the secondary outcome paresthesia (for which the sample size was not calculated) with no mention of the main purpose of the study (for which the sample size was calculated). This should be rewritten.
8. References:
   a. Some have DOI while some do not. Please be consistent.
   b. Ref# 2, 3 and 17 have no year of publication. The authors have mentioned n.d. that I presume is ‘no date’. This is incorrect. Please go on pubmed to find the correct year.
   c. Ref#18 has a special symbol the purpose of which I cannot understand.
