# Supplementary material for: Extraplexus versus intraplexus ultrasound-guided interscalene brachial plexus block for ambulatory arthroscopic shoulder surgery: A randomized controlled trial
Source: PLoS One. 2021 Feb 18;16(2):e0246792. doi: 10.1371/journal.pone.0246792 (PMC7891753; doi:10.1371/journal.pone.0246792)
Supplement: Attachment — Submitted filename: Response to Reviewers.docx [file pone.0246792.s008.docx]

January 9, 2021

Re: PONE-D-20-12274 “Extraplexus versus intraplexus ultrasound-guided interscalene brachial plexus block for ambulatory arthroscopic shoulder surgery: a randomized controlled trial”

Dear Editor Johannes Fleckenstein,

Thank you for the opportunity to revise our manuscript PONE-D-20-12274 titled, “Extraplexus versus intraplexus ultrasound-guided interscalene brachial plexus block for ambulatory arthroscopic shoulder surgery: a randomized controlled trial.” We appreciate the reviewers’ comments and have incorporated their feedback. We have addressed each comment in blue font below. We believe that this has greatly improved the manuscript and thank you for considering our revised manuscript.

Sincerely,

Monica W. Harbell, MD

Reviewer #2 comments:

1. This is an old study. As the authors mention, it was registered in 2012 and the results were presented in 2016.

- While a few studies have investigated intraplexus vs. extraplexus interscalene blocks in the past, we believe that this current study adds to the body of literature by providing a more detailed depiction of motor and sensory loss after interscalene block. Further, previous studies have not looked into the safety outcomes of these two approaches.

2. The authors have used a large volume of local anesthetic for the block. They do give a valid reason.

- We thank the reviewer for understanding why the volume used in this study was chosen.

3. Sample size
   a. Sample size was not adjusted for dropouts. This always leads to problems when there is missing data, postoperatively. In this study, the sample size for the primary objective was 104 per group. In statistical sense, authors did not reach the sample size that they were supposed to. They only had 103 patients in one group and though the difference is only 1, mathematically and statistically, they did not attain the calculated sample. This problem would not have happened if they had increased their sample by 10% (or any number, for that matter).

- Sample size calculations for survival data (Freedman method) differ from the usual sample size calculation. It is a composite of two steps: 1. Calculation of the number of events needed to show a statistically significant difference (191 events = number patients with loss of shoulder abduction during our study period). 2. Adjustment of the sample size for administrative censoring. Those are patients that never have an event (sample size increase of 17 to account for possible patients with no loss of shoulder abduction during our study period).
- In our study, it turned out that all of our patients in the study lost shoulder abduction. The number of events for the primary outcome was therefore 208, far exceeding the required number of events of 191. It also turned out that we did not have any dropouts for the primary outcome. All 208 patients were included in the analysis of time to shoulder abduction. In a statistical and mathematical sense, the sample size in our study is sufficient.
  b. The authors should explain why there was unequal distribution of patients across the 2 groups resulting in inadequate sample in the intraplexus group (105 vs 103).
- This is the effect of the simple randomization scheme we chose for our study. This is a well-accepted type of randomization in the scientific community. The uneven numbers between groups are likely to occur with this randomization scheme and are only problematic with small sample sizes (less than 20). In a statistical and mathematical sense, the addition or subtraction of one patient in one group or the other will not affect the results in a trial with a sample size of over 200 participants.
  c. The primary outcome was time to block and the sample size was calculated for this outcome only. Paresthesia is the only secondary outcome significant finding (other than the primary outcome). However, it was a secondary outcome and the sample was not calculated for this outcome. This should be mentioned in the limitations.
- It is common in clinical research that the sample size is only calculated for the primary outcome. The reviewer is correct that we cannot conclude that the non-significant secondary outcomes indicate no difference between study groups. However, when there is a significant statistical difference in a secondary outcome, it is not expected that the difference will go away with increasing sample size. It is therefore legitimate to conclude that there was a significant difference in paresthesia between study groups as we have done in this study.

4. In the extraplexus group, one patient was lost to followup but the n in the tables remains 105. Similarly, there were 2 patients in the intraplexus group, but the n remains 103.

- In the extraplexus group, one patient was excluded from part of the sensory exam analysis as they were missing sensory data for that particular nerve distribution. This patient was included in the rest of the analysis (motor block, POD#1 questions, etc). A different patient in the extraplexus group who was unable to be reached for the POD#1 call was excluded for the POD#1 questions, but all the remaining data was able to be analyzed for that group. Similarly, in the intraplexus group, it was not the same patient was missing both sensory and POD#1 data. The tables contain a mix of data from the day of surgery and POD#1. For this reason, the n remains 105 in the extraplexus group and 103 in the intraplexus group in the tables.

5. Unblinding is a very big limitation in a double-blind trial, however small the percentage may be. It would be best to use single blind and remove the sections explaining 10% blinding and lack of staffing. Lack of staffing is a local matter that everyone faces but never acknowledges in scientific studies. For most ethics review boards, one has to ascertain that they have enough staff, resources and funding to be able to do the study. Therefore, one cannot (should not) say that during the study, they did not have enough staff.

- We appreciate the Reviewer’s comments and revised the manuscript to remove mention of lack of staffing. Given that only a small proportion of the participants experienced unblinding for sensory and motor assessments, we believe that the most accurate way to describe the study is as a double blinded study and we believe that ethically we should disclose that 10% were unblinded.

6. Paresthesia is an important secondary outcome of this study. The authors should spend a few lines describing what they considered paresthesia in the context of this work.

- We thank the reviewer for this suggestion. We have added a few lines describing what was defined as paresthesia to the Methods section.

7. Conclusions: The conclusions focus on the secondary outcome paresthesia (for which the sample size was not calculated) with no mention of the main purpose of the study (for which the sample size was calculated). This should be rewritten.

- We have emphasized the primary outcome of this study in the revised version of the Conclusion.

8. References:
   a. Some have DOI while some do not. Please be consistent.
   b. Ref# 2, 3 and 17 have no year of publication. The authors have mentioned n.d. that I presume is ‘no date’. This is incorrect. Please go on pubmed to find the correct year.
   c. Ref#18 has a special symbol the purpose of which I cannot understand.

- Our most recent version of the manuscript has 18 references. We have updated and corrected the references with the suggested changes.
